# Impostor phenomenon is a common feature among individuals with primary hyperhidrosis

**DOI:** 10.1177/20503121231220828

**Published:** 2024-01-27

**Authors:** Alexander Shayesteh, Jens Boman, Elisabet Nylander

**Affiliations:** 1 Department of Public Health and Clinical Medicine, Dermatology and Venereology, Umeå University, Umeå, Sweden; 2 Department of Clinical Science, Professional Development, Umeå University, Umeå, Sweden

**Keywords:** Primary hyperhidrosis, impostor phenomenon, perfectionism, self-compassion, questionnaires

## Abstract

**Background:**

Primary hyperhidrosis consists of excessive focal sweating. Affected individuals camouflage the sweating on their body, avoiding stigmatisation. Hence, misrepresentation in social interactions is a common feature in patients with hyperhidrosis. The aim of this study was to investigate impostor phenomenon, perfectionism, self-compassion, stress and anxiety among individuals with primary hyperhidrosis.

**Methods:**

A cross-sectional study was conducted at our clinic among 100 participants with axillary and palmar primary hyperhidrosis. The questionnaire contained a hyperhidrosis part and Perceived Stress Scale-4, Generalised Anxiety Disorder Scale-2, Clinical Perfectionism Questionnaire-6, Self-Compassion Scale Short form and Clance Impostor Phenomenon Scale. Descriptive statistics was used for analyses of categorical variables. As data were normally distributed independent *t*-test and one-way analysis of variance with post hoc Tukey test were used to compare the mean values for the questionnaires with other variables. Pearson’s correlation was used, and a forward multiple linear regression model was performed to predict presence of impostor phenomenon with gender, age and other scales in this study.

**Results:**

Impostor phenomenon occurred in almost half of our patients (48%) with hyperhidrosis. While feelings of impostor phenomenon were more common in women, there was no difference between gender regarding its intensity levels (*p* = 0.07). In addition, we found a significant (*p* < 0.001) negative correlation between impostor phenomenon and self-compassion, while feelings of impostoer phenomenon increased with stress, anxiety and perfectionism (*p* < 0.001).

**Conclusions:**

Feelings of impostor phenomenon was found in 48% of individuals with hyperhidrosis which indicates that it is a common feature in this patient group. Future research is warranted regarding the prevalence of impostor phenomenon in hyperhidrosis and other medical conditions, among men and women, seeking medical healthcare. Psychological interventions in hyperhidrosis may be beneficial both for the individual and in public health, by facilitating management of patients’ daily lives and saving considerable resources in healthcare regarding pharmacological interventions and medical consultations.

## Introduction

Primary hyperhidrosis (PH) means excessive and focal sweating affecting 5.5% of the population in Sweden.^1^ Sweating in PH is often initiated in episodes or at rest, exacerbated by emotional factors. The most common localisation for PH is the axillary region affecting men and women equally.^2^ The diagnosis of PH is set due to medical history, and there are no objective or simple methods for assessment of excessive sweat in clinical practice.^3^ Hyperhidrosis Disease Severity Scale (HDSS) is a short and validated scale used by patients for assessing the severity of hyperhidrosis. The HDSS is composed of one question with four answers. One to two points indicate mild or moderate hyperhidrosis while three to four points indicate severe sweating problems.^3,4^

The excessive sweat in PH interferes in daily interactions and reduces quality of life significantly in comparison with other dermatological diseases.^5^ In addition, hyperhidrosis has negative effects on the individual’s economy as clothes are often worn out; in private life from difficulties in finding acceptance and support from others; in occupational life such as the impact on physical tasks and the ability to wear uniforms; and in social life with the risk of being subject to the surrounding’s prejudice of being nervous or unclean.^6,7^ These factors have been described as contributing to withdrawal, and signs of anxiety and depression in those affected.^8,9^ The stress and anxiety of being noticed by others for having excessive sweating could more often affect perfectionist individuals as obsessive measures, for maintaining unrealistic standards, stimulates the autonomic nervous system thus, exacerbating or triggering hyperhidrosis.

Impostor phenomenon (IP) has been described to affect high-achieving individuals who, despite their objective successes, have persistent self-doubt and fear of being exposed as a fraud.^10^ There is no reliable data regarding the natural progression of IP with increasing age. However, as IP could facilitate the belief in one’s abilities to achieve certain results, it may also reduce mental health leading to withdrawal, stigmatisation and self-isolation.^11^ While IP is not a diagnosis or a psychiatric disorder, symptoms from IP could be a comorbid finding in individuals with depression and anxiety.^12^ Many attributes reported in IP have also commonly been described in patients suffering from PH. Since no objective methods exist in clinical practice to assess the quantity of sweat, excessive sweating is often considered a relative term. In hyperhidrosis, IP could be a potentially exacerbating psychological condition, challenging one’s self-view regarding physical abilities and social desirability. Thus, the aim of this study was to investigate the prevalence and severity of IP, perfectionism, self-compassion, stress and anxiety in patients with PH.

## Methods

### Study design

A cross-sectional survey with questionnaires was conducted in patients with axillary and palmar hyperhidrosis.

### Procedure and participants

Patients of 18 years of age, attending the hyperhidrosis section of the Department of Dermatology and Venereology, University Hospital, Umeå, Sweden between June 2022 and April 2023 were consecutively included in the study. All patients had previously used topical treatments without satisfactory effect, and they all had axillary and/or palmar PH. The participants had obtained the diagnosis of PH from a general practitioner, and the diagnosis was confirmed by a dermatologist on the day of the visit. Exclusion criteria were pregnancy, lactation, secondary hyperhidrosis and treatment with botulinum toxin within a year prior to the study. Participants were asked for consent and received the questionnaires on the day of their visit from healthcare personnel other than their physician. The responses to the coded questionnaires were collected via a drop-box situated in the waiting room.

### Materials for data collection

The background questionnaire included a socio-demographic section investigating gender and age, the latter stratified into seven age categories (⩽24, 25–29, 30–34, 35–39, 40–44, 45–49, ⩾50). The clinical variables included localisation of hyperhidrosis according to palmar or axillary, and severity of hyperhidrosis among other factors. In addition to the background questionnaire, short forms of five validated psychometric instruments, investigating signs of stress (Perceived Stress Scale-4), anxiety (Generalised Anxiety Disorder Scale-2), perfectionism (Clinical Perfectionism Questionnaire-6), self-compassion (Self-Compassion Scale Short Form) and IP (Clance Impostor Phenomenon Scale) were added. All questionnaires were free for use in research and available in the Swedish language (Supplemental file 1).

### Psychometric instruments

#### Perceived Stress Scale-4

Perceived Stress Scale-4 (PSS-4) measures the level of stress and consists of four questions using a 5-point (p) Likert scale (4-p for ‘very often’ to 0-p for ‘never’). PSS-4 yields a maximum score of 16 p; 0–8 p interpreted as normal stress levels and 9–16 p indicating an elevated stress level.^13^ PSS-4 was tested in an English sample of 1568 individuals and described as having acceptable psychometric properties (Cronbach α = 0.77).^13^ The Swedish version of PSS-4 has been described measuring the general, rather than specific aspects of stress.^14^

#### Generalised Anxiety Disorder Scale-2

Generalised Anxiety Disorder Scale-2 (GAD-2) is a shorter validated form of Generalised Anxiety Disorder Scale-7 (GAD-7), which aims to find those at risk of anxiety disorder. GAD-2 consists of two questions with a 4-p Likert scale answers (3-p for ‘nearly every day’ to 0-p for ‘not at all’). In GAD-2 a score of 0-2 p indicates normal levels of anxiety, whereas 3–6 p represents an elevated risk for anxiety disorders.^15^ The GAD-2 questionnaire has been validated in multiple studies and shown to retain the excellent psychometric properties of GAD-7 (Cronbach α = 0.92 and intraclass correlation = 0.83). Thus, GAD-2 has been proposed as an essential first step for screening generalised anxiety disorder.^15^

#### Clinical Perfectionism Questionnaire-6

The Clinical Perfectionism Questionnaire-6 (CPQ-6) assesses the level of perfectionism by investigating strives to meet standards and effects, on self-evaluation when standards are not met.^16^ CPQ-6 consists of six questions, using a 4-p Likert scale (4-p for always to 1-p for never), yielding a total score of 6–24 p; 6–10 p as ‘no problems,’ 11–15 p as ‘moderate problems’, 16–20 p as ‘difficult problems’ and 21–24 p as ‘severe’ problems with perfectionism.^17^ The Swedish version of CPQ-6 was described having a test-retest correlation of *r* = 0.62 and an internal consistency of Cronbach α = 0.72.^17^

#### Self-Compassion Scale Short Form

Self-compassion has been associated with psychological well-being and is an important protective factor that fosters emotional resilience. A short and validated version of the original Self-Compassion Scale, Self-Compassion Scale Short Form (SCS-SF) consists of 12 questions, with a corresponding Likert scale of 5 alternative answers (5-p for ‘almost always’ to 1-p for ‘almost never’).^18^ The total score for all questions in SCS-SF is further used to calculate a mean score; 1.00–2.49 p indicating ‘low’ self-compassion, 2.50–3.50 p ‘moderate’ and 3.51–5.00 p ‘high’ self-compassion.^19^ SCS-SF has been described having a good internal consistency (Cronbach α = 0.85) and correlates well with other self-reported measures.^19^

#### Clance Impostor Phenomenon Scale

Clance Impostor Phenomenon Scale (CIPS) is a validated scale measuring the level of IP.^20,21^ It was translated to Swedish from English followed by retranslation to English by two professional translators, and the English backtranslation was then approved by Dr. Clance. CIPS consists of 20 questions, each with a 5-p Likert scale (5-p for ‘very true’ to 1-p for ‘not at all true’) resulting in a total score of 20–100 p. CIPS scoring system can be described as 20–40 p as ‘having few’, 41–60 p as ‘having moderate’, 61–80 p as ‘having frequent’ and 81–100 p as ‘having intense’ IP feelings. The scoring system of the CIPS questionnaire has been further defined by Holmes et al.,^21^ defining a cut-off ⩾62-p on the scale, which would represent IP. Although CIPS is not validated in Swedish, it has been used in a small study among 30 patients with provoked vulvodynia.^22^

### Ethical considerations

This study was approved by the Ethical Review Board in Sweden, Dnr. 2022-01511-01. Data were collected after each participant provided written informed consent. Data were kept confidential with only the research team having access to it. There were no risks involved in participating in this study.

#### Statistics

##### Sample size and sampling technique

A total of 100 persons with PH was considered a reasonable sample size, enabling statistical tests of difference within this sample. A convenience sampling technique was used due to the scarcity of hyperhidrosis and no access to prior data regarding the aims of this study. A prior precision calculation using Cochran’s equation together with a population correction to calculate was considered to have; a precision level ±15%, (a result within 15 percentage points of the true population value), unknown prior knowledge about IP within the population contributing to a variability of 0.5 (0–1), and a confidence interval of 95% within a selected population of 100 individuals, yielding a sample size of *n* = 31 with IP, needed for this study.

##### Data analyses

Descriptive statistics and chi-square test was used to measure proportional differences for categorical variables. Independent *t*-test was used for calculating differences in mean data between gender and the scales used in this study. One-way ANOVA with post hoc Tukey analysis was used to investigate significant differences in age groups with mean data regarding PSS-4, GAD-2, CPQ-6, SCS-SF and CIPS. Pearson’s correlation was used to investigate the correlation between CIPS scores with PSS-4, GAD-2, CPQ-6 and SCS-SF. Pearson’s correlation was also used for relating the severity of hyperhidrosis (HDSS) to age, gender, CIPS, PSS-4, GAD-2, CPQ-6 and SCS-SF.

A forward multiple linear regression model was constructed to predict CIPS score as the dependent variable, tested with independent variables; gender, age, HDSS, PSS-4, GAD-2, CPQ-6 and SCS-SF. SPSS Statistics version 25 (IBM Corp; Amonk, NY, USA). was used for calculations and data analysis. The level of significance was set at *p* < 0.05.

## Results

A total of 100 individuals (38 men, 62 women) participated. No missing data was found. The most common age category reported by the participants was 18–24 years (39%), axillary hyperhidrosis was reported by 69% and palmar hyperhidrosis by 31%. Chi-test resulted in no significant difference in background characteristics between men and women, presented in Table 1.

**Table 1.** Background characteristics of the participants.

Variables	All	Men	Women
Number	100	38	62
Age (%)			
18–24	39	12 (32)	27 (44)
25–29	20	8 (21)	12 (20)
30–34	14	7 (18)	7 (11)
35–39	13	5 (13)	8 (13)
40–44	5	1 (3)	4 (7)
45–49	3	2 (5)	1 (2)
⩾50	6	3 (8)	3 (5)
Hyperhidrosis (%)			
Axillary	69	28 (74)	41 (66)
Palmar	31	10 (26)	21 (34)
VAS-10 p, mean ± SD	7.88 ± 1.18	7.74 ± 1.20	7.96 ± 1.17
HDSS, mean ± SD	3.0 ± 0.78	3.0 ± 0.87	3.0 ± 0.72

HDSS: hyperhidrosis disease severity scale; VAS; visual analogue scale; SD; standard deviation; p: points.

Mean score data of PSS-4, GAD-2, CPQ-6, SCS-SF and CIPS scales for individuals with PH are described in Table 2.

**Table 2.** PSS-4, GAD-2, CPQ-6, SCS-SF and CIPS mean scores in patients with PH.

Psychometric instruments	All (*N* = 100)	Men (*N* = 38)	Women (*N* = 62)
PSS-4, mean ± SD	5.68 ± 2.57	5.42 ± 3.78	5.84 ± 2.69
GAD-2, mean ± SD	1.94 ± 1.78	1.21 ± 1.07	2.40 ± 1.98*
CPQ-6, mean ± SD	15.06 ± 4.31	13.97 ± 4.17	15.73 ± 4.29
SCS-SF, mean ± SD	3.18 ± 0.70	3.32 ± 0.64	3.09 ± 0.73
CIPS, mean ± SD	59.1 ± 17.2	55.1 ± 16.8	61.5 ± 17.1

N: number; PSS-4: Perceived Stress Scale-4; GAD-2: Generalised Anxiety Disorder-2; CPQ-6: Clinical Perfectionism Questionnaire-6; SCS-SF: Self-Compassion Scale Short Form; CIPS: Clance Impostor Phenomenon Scale; SD: standard deviation.

* *p* < 0.05, **p < 0.01, ***p < 0.001.

### Stress and anxiety (PSS-4 and GAD-2)

In total 14% (13.1% men and 14.5% women) had a score above 8 points on the PSS-4 scale, indicating elevated levels of stress. However, there was no significant difference in gender regarding the prevalence of stress *p* = 0.62. In GAD-2, a total of 29/100 individuals had a score of ⩾3 points indicating signs of anxiety. This group consisted of 89.7% (26/29) women and 10.3% (3/29) men (*p* < 0.001). Individuals with axillary hyperhidrosis had high GAD-2 mean points (*p* = 0.02). One-way ANOVA-test and POST HOC analysis with Tukey for age groups, resulted in a significant decrease (*p* < 0.01) for both PSS-4 and GAD-2 scale means scores, with increasing age.

### Perfectionism (CPQ-6)

According to CPQ-6, a total of 17/100 individuals reported limited problems (6–10 points) with perfectionism, 32/100 of them had moderate problems (11–15 points), 44/100 of them had severe problems (16–20 points) and 7/100 of them had extremely severe problems (21–24 points). There was no significant difference between men and women (*p* = 0.97) or between age groups (*p* = 0.059) for mean CPQ-6 scores.

### Self-compassion (SCS-SF)

Low self-compassion (1.0–2.49 SCS-SF mean points) was reported by 16/100, moderate (2.50–3.50 points) by 54/100 and high level (3.51–5.0) by 30/100 individuals. The SCS-SF mean points for self-compassion differed between age groups, increasing with higher age (*p* < 0.001) but not between men and women (*p* = 0.33) or with other background variables such as VAS, HDSS, and localisation of sweating.

### Impostor phenomenon

**Figure 1.**
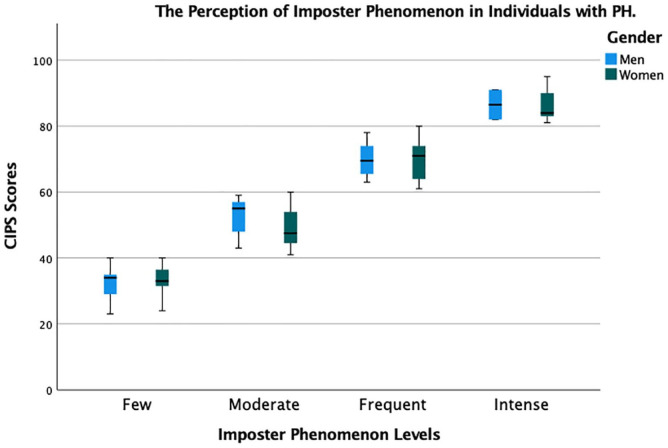
The perception of IP levels in individuals with PH. Impostor levels as few feelings (20–40 points, *n* = 16), moderate feelings (41–60 points, *n* = 35), frequent feelings (61–81 points, *n* = 38) and intense feelings (81–100 points, *n* = 11). There was no significant difference between men and women for impostor mean scores according to CIPS, (*p* = 0.07).

IP (CIPS score ⩾62-p) was present in 48/100 participants (34 women and 14 men) (Figure 1). There was no significant difference for CIPS regarding the severity of hyperhidrosis, according to HDSS (*p* = 0.18), or localisation (*p* = 0.76). However, among the age groups, CIPS mean scores were significantly reduced with increasing age (*p* < 0.001) (⩾50 years, 39.8 ± 10.2; ⩽24 years, 65.5 ± 12.5).

A significant negative correlation was found between IP and self-compassion, whereas there was a positive correlation between IP and stress, anxiety and perfectionism (Table 3). There was no significant correlation between the severity of hyperhidrosis (HDSS) and gender, age, PSS-4, GAD-2, CPQ-6, SCS-SF and CIPS mean scores.

**Table 3.** Correlations between the score of CIPS, SCS-SF., PSS-4, GAD-2 and CPQ-6, respectively.

Impostor phenomenon	PSS-4		GAD-2		CPQ-6		SCS-SF	
	*r*	*p*	*r*	*p*	*r*	*p*	*r*	*P*
CIPS	0.45	<0.001	0.47	<0.001	0.32	<0.001	−0.62	<0.001

CIPS: Clance Impostor Phenomenon Scale; PSS-4: Perceived Stress Scale-4; GAD-2: Generalised Anxiety Disorder-2; CPQ-6: Clinical Perfectionism Questionnaire-6; SCS-SF: Self-Compassion Scale Short Form; r: Pearson’s correlation coefficient.

A forward multiple linear regression model of IP as CIPS (dependent variable) and gender, age, HDSS, PSS-4, GAD-2, CPQ-6 and SCS-SF (independent variables) (Table 4) resulted in perfectionism being the strongest predictor for IP followed by low self-compassion and age, all accounting for 56.5% of the variation (*R*^2^ = 0.565, *p* = 0.036).

**Table 4.** Hierarchical step by step regression analysis predicting CIPS.

Step	Variable(s)	B	*R*^2^ change	F	95 % CI for B	
Outcome variable = CIPS						
1	CPQ–6	2.710***	0.459	83.270***	2.121	3.299
2	CPQ–6	1.974***			1.352	2.595
	SCS-SF	−0.742***	0.549	21.577***	−1.052	−0.425
3	CPQ–6	1.923***			1.311	2.535
	SCS-SF	−0.609***			−0.944	−0.274
	Age	−1.534*	0.565	4.539*	−2.964	−0.105

CIPS: Clance impostor phenomenon scale; CPQ-6: clinical perfectionism questionnaire-6; SCS-SF: self-compassion scale short form.

* *p* < 0.05, **p < 0.01, ***p < 0.001.

## Discussions

This is, to our knowledge, the first study evaluating the occurrence of IP in individuals with PH. We found that almost half of the patients (48%) with PH had signs of IP, with a majority (70%) being women. Despite the female overrepresentation, we found no difference between men and women regarding the intensity levels (48%) of IP experiences (*p* = 0.07) but increasing age significantly reduced (*p* < 0.001) the mean scores of CIPS testing for IP.

Former studies investigating the prevalence of PH have not found any gender differences in the population;^1,2^ however, women with hyperhidrosis more often seek medical care. It would, thus, be of importance to consider the occurrence of IP in women with hyperhidrosis, as one factor causing increased need for medical attention. Evaluating IP by gender is often reported among healthy persons without known diseases, and the results have yet been inconclusive. A systematic review of 33 studies found no increased IP rates in women in 17 out of the 33 studies investigated.^12^ Thus, much more research is needed for investigating the role of IP and gender, and IP in stigmatising diseases such as hyperhidrosis.

The prevalence of IP among individuals with PH could be challenging to interpret. It is often described that the occurrence of IP may vary depending on recruitment, screening tools and the population investigated. The review by Bravata et al.^12^ found an IP prevalence of 9%–82% in 62 studies, suggesting a publication bias for higher prevalence numbers. Hence, the prevalence of IP according to our study setting, has to be contextualised. A common and effective, but expensive treatment for PH consists of repeated injections with botulinum toxin.^23^ A challenge, often described by clinicians, is to assess the need for re-treatments, in patients seeking healthcare while scoring low on HDSS without visual sweating. There is no consensus on how to manage hyperhidrosis patients without objective signs; however, our clinical experience is that most clinicians repeat treatment.

Another interesting and related issue is that low self-compassion has also been associated with a decrease in health-promoting behaviours such as mindfulness, self-care and self-efficacy.^24^ In a disease such as diabetes, increased self-compassion has been described to improve levels of distress/ depression, interpersonal communication and psychological inflexibility.^24^ Considering all these facts, we believe that there is a role for psychological intervention for patients with PH as well. A psychological intervention would address several aspects of daily life such as: (a) empowering the patients to accept their disease and finding tools for managing fear from situations which often exacerbate sweat production, (b) engaging in forums with others in the same situation to discuss and validate their struggles from hyperhidrosis, (c) combat their feelings of perfectionism, not being good enough in social life, (d) exercising self-compassion instead of focusing on external norms and expectations and finally, (e) becoming aware of self-feelings and fears, rather than continuously adapting to the surrounding society, which is described to hinder and restricts daily life activities. Thus, acceptance, awareness, self-compassion and coping tools to manage psychological impairments associated with hyperhidrosis, could help those affected to obtain a better quality of life and reduce the dependency of repeated and avoidable medical treatments. Our impression of an intervention in healthy individuals with high scores on CIPS indicates that a short intervention during 5 weeks may reduce feelings of IP (pers comm). This measure could potentially have several public health implications such as increasing social and private interactions with others, increasing productivity at work and gains in quality of life by strengthening the individual’s ability of self-care and increasing the individual’s resilience against negative attributions associated with hyperhidrosis. Future research will show if such an intervention among hyperhidrosis patients, could improve both psycho-social health and diminish the need for repeated treatments.

### Strengths and limitations

Assessment of causality was not possible in this cross-sectional setting; hence, some care must be taken when interpreting our findings and it would be difficult to generalise our results to other diseases. However, as there is no previous data regarding IP in hyperhidrosis, our findings will further contribute to the psychopathology associated with the disease and could enable further research in addressing the mental health of this patient group.

Another limitation could be that we did not specifically investigate psychological comorbidities among the study participants. While psychological comorbidities could have been present among our participants, the diagnosis of PH should not have an apparent cause. Since participants did not report having other comorbidities, and PH was diagnosed both by a GP and a dermatologist, it is reasonable to assume that unknown comorbidities could not have had a significant effect on our findings.

Also, as women constituted most of our participants, our findings could have been affected by both biological and non-biological explanations. Population studies regarding hyperhidrosis often report no gender difference regarding the prevalence of PH. As the complexity of IP is not well understood, more research is needed to examine the impacts of IP in men and women, as it could confirm our findings and facilitate knowledge in this field of research.

## Conclusions

Feelings of IP was found in 48% of individuals with hyperhidrosis which indicates that it is a common feature in this patient group. Future research is warranted regarding the prevalence of IP in hyperhidrosis and other medical conditions, among men and women, seeking medical healthcare. Psychological interventions in hyperhidrosis, may be beneficial both for the individual and in public health, by facilitating management of patients’ daily lives and saving considerable resources in healthcare regarding pharmacological interventions and medical consultations.

## Supplemental Material

sj-docx-1-smo-10.1177_20503121231220828 – Supplemental material for Impostor phenomenon is a common feature among individuals with primary hyperhidrosis Click here for additional data file. Supplemental material, sj-docx-1-smo-10.1177_20503121231220828 for Impostor phenomenon is a common feature among individuals with primary hyperhidrosis by Alexander Shayesteh, Jens Boman and Elisabet Nylander in SAGE Open Medicine
