# Supplementary material for: Impostor phenomenon is a common feature among individuals with primary hyperhidrosis
Source: SAGE Open Med. 2024 Jan 27;12:20503121231220828. doi: 10.1177/20503121231220828 (PMC10822058; doi:10.1177/20503121231220828)
Supplement: sj-docx-1-smo-10.1177_20503121231220828 – Supplemental material for Impostor phenomenon is a common feature among individuals with primary hyperhidrosis [file sj-docx-1-smo-10.1177_20503121231220828.docx]

Code:……. Date:……………………

**Part I. Background questions**

1. My gender is □ male □ female □ other

2. How old are you (years)?

□ 24 or below □ 25-29 □ 30-34 □ 35-39 □ 40-44 □ 45-49 □ 50+

3. Where on your body do you suffer from excessive sweating? (choose one option)

□ feet □ palms □ axilla □ face □ other____________________

4. How would you rate the severity of your sweating according to the VAS scale?

0 1 2 3 4 5 6 7 8 9 10

No symptoms
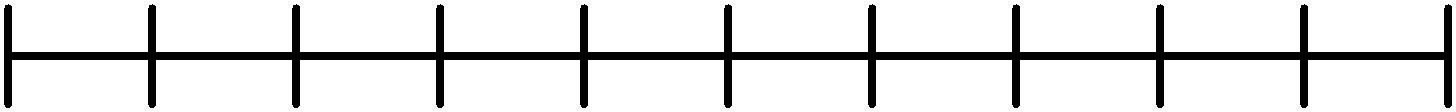
 Intolerable

5. How would you rate the severity of your hyperhidrosis according to HDSS?

□ My sweating is never noticeable and never interferes with my daily activities

□ My sweating is tolerable but sometimes interferes with my daily activities

□ My sweating is barely tolerable and frequently interferes with my daily activities

□ My sweating is intolerable and always interferes with my daily activities

**Part II. Perceived Stress Scale-4 (PSS-4)**

*These questions concerns your feelings and thoughts during THE LAST MONTH. In each case, please indicate your response by placing an “X” over the square representing HOW OFTEN you felt in a certain way.*

6. In the last month, how often have you felt that you were unable to control the important things in your life?

□ never □ almost never □ sometimes □ fairly often □ very often

7. In the last month, how often have you felt confident about your ability to handle your personal problems?

□ never □ almost never □ sometimes □ fairly often □ very often

8. In the last month, how often have you felt that things were going your way?

□ never □ almost never □ sometimes □ fairly often □ very often

9. In the last month, how often have you felt difficulties were piling up so high that you could not overcome them?

□ never □ almost never □ sometimes □ fairly often □ very often

**Part III. Generalized Anxiety Disorder Scale-2 (GAD-2)**

Over the last 2 weeks, how often have you been bothered by the following problems?

10. Feeling nervous, anxious, or on edge

□ not at all □ several days □ more than half the days □ nearly every day

11. Not being able to stop or control worrying

□ not at all □ several days □ more than half the days □ nearly every day

**Part IV. Self-Compassion Scale Short form (SCS-SF)**

*Please read each statement carefully before answering. Indicate how often you behave in the stated manner, using the following scale*

12. When I fail at something important to me I become consumed by feelings of inadequacy.

□ almost never □ seldom □ sometimes □ often □ almost always

13. I try to be understanding and patient towards those aspects of my personality I don’t like.

□ almost never □ seldom □ sometimes □ often □ almost always

14.When something painful happens I try to take a balanced view of the situation.

□ almost never □ seldom □ sometimes □ often □ almost always

15. When I’m feeling down, I tend to feel like most other people are probably happier than I am.

□ almost never □ seldom □ sometimes □ often □ almost always

16. I try to see my failings as part of the human condition.

□ almost never □ seldom □ sometimes □ often □ almost always

17. When I’m going through a very hard time, I give myself the caring and tenderness I need.

□ almost never □ seldom □ sometimes □ often □ almost always

18. When something upsets me I try to keep my emotions in balance.

□ almost never □ seldom □ sometimes □ often □ almost always

19. When I fail at something that’s important to me, I tend to feel alone in my failure.

□ almost never □ seldom □ sometimes □ often □ almost always

20. When I’m feeling down I tend to obsess and fixate on everything that’s wrong.

□ almost never □ seldom □ sometimes □ often □ almost always

21. When I feel inadequate in some way, I try to remind myself that feelings of inadequacy are shared by most people.

□ almost never □ seldom □ sometimes □ often □ almost always

22. I’m disapproving and judgmental about my own flaws and inadequacies.

□ almost never □ rarely □ sometimes □ often □ almost always

23. I’m intolerant and impatient towards those aspects of my personality I don’t like

□ almost never □ rarely □ sometimes □ often □ almost always

**Part V. Clinical Perfectionism Questionnaire-6 (CPQ-6)**

*Questions below concern perfectionism. Perfectionism is defined as striving to meet high self-constructed demands, regardless of being successful in achieving them or not. Focus for these questions is only perfectionism regarding other life-experiences than food intake, weight and body image.*

*Over the past month,…*

24. Have you pushed yourself really hard to meet your goals?

□ not at all □ rarely □ sometimes □ all the time

25. Have you been told that your standards are too high?

□ not at all □ rarely □ sometimes □ all the time

26. Have you felt a failure as a person because you have not succeeded at meeting your goals?

□ not at all □ rarely □ sometimes □ all the time

27. Have you been afraid that you might not reach your standards?

□ not at all □ rarely □ sometimes □ all the time

28. Have you judged yourself on the basis of your ability to achieve high standards?

□ not at all □ rarely □ sometimes □ all the time

29. Have you kept trying to meet your standards, even if this has meant that you have missed out on things?

□ not at all □ rarely □ sometimes □ all the time

**Part VI.** **Clance Impostor Phenomenon Scale (CIPS)**

*For each question, please circle the number that best indicates how true the statement is for you. Please give the first response that enters your mind rather than dwelling on each statement and thinking about it over and over. The scales are from 1-5; 1= not at all true, 2 = rarely, 3 = sometimes, 4 = often, 5 = very true*

30. I have often succeeded on a test or task even though I was afraid that I would not do well before I undertook the task.

1 2 3 4 5

31. I can give the impression that I’m more competent than I really am.

1 2 3 4 5

32. I avoid evaluations if possible and have a dread of others evaluating me.

1 2 3 4 5

33. When people praise me for something I’ve accomplished, I’m afraid I won’t be able to live up to their expectations of me in the future.

1 2 3 4 5

34. I sometimes think I obtained my present position or gained my present success because I happened to be in the right place at the right time or knew the right people.

1 2 3 4 5

35. I’m afraid people important to me may find out that I’m not as capable as they think I am.

1 2 3 4 5

36. I tend to remember the incidents in which I have not done my best more than those times I have done my best.

1 2 3 4 5

37. I rarely do a project or task as well as I’d like to do it.

1 2 3 4 5

38. Sometimes I feel or believe that my success in my life or in my job has been the result of some kind of error.

1 2 3 4 5

39. It’s hard for me to accept compliments or praise about my intelligence or accomplishments.

1 2 3 4 5

40. At times, I feel my success has been due to some kind of luck

1 2 3 4 5

41. I’m disappointed at times in my present accomplishments and think I should have accomplished much more.

1 2 3 4 5

42. Sometimes I’m afraid others will discover how much knowledge or ability I really lack.

1 2 3 4 5

43. I’m often afraid that I may fail at a new assignment or undertaking even though I generally do well at what I attempt.

1 2 3 4 5

44. When I’ve succeeded at something and received recognition for my accomplishments, I have doubts that I can keep repeating that success.

1 2 3 4 5

45. If I receive a great deal of praise and recognition for something I’ve accomplished, I tend to discount the importance of what I’ve done.

1 2 3 4 5

46. I often compare my ability to those around me and think they may be more intelligent than I am.

1 2 3 4 5

47. I often worry about not succeeding with a project or examination, even though others around me have considerable confidence that I will do well.

1 2 3 4 5

48. If I’m going to receive a promotion or gain recognition of some kind, I hesitate to tell others until it is an accomplished fact.

1 2 3 4 5

49. I feel bad and discouraged if I’m not “the best” or at least “very special” in situations that involve achievement.

1 2 3 4 5

**Thank you for answering our questionnaire**
